# Hydrogen-Bonded Cyclic Dimers at Large Compression: The Case of 1*H*-pyrrolo[3,2-*h*]quinoline and 2-(2′-pyridyl)pyrrole

**DOI:** 10.3390/molecules26133802

**Published:** 2021-06-22

**Authors:** Dominik Kurzydłowski, Taisiia Chumak, Jakub Rogoża, Arkadiusz Listkowski

**Affiliations:** 1 Faculty of Mathematics and Natural Sciences, Cardinal Stefan Wyszyński University, 01-038 Warsaw, Poland; taisiia.chumak@gmail.com (T.C.); a.listkowski@uksw.edu.pl (A.L.); 2 Faculty of Physics, University of Warsaw, 02-093 Warsaw, Poland; jakub.rogoza@fuw.edu.pl; 3 Institute of Physical Chemistry, Polish Academy of Sciences, 01-224 Warsaw, Poland

**Keywords:** proton transfer, hydrogen bond, polymorphism, high pressure, diamond anvil cell

## Abstract

1*H*-pyrrolo[3,2-*h*]qinoline (PQ) and 2-(2′-pyridyl)pyrrole (PP) are important systems in the study of proton-transfer reactions. These molecules possess hydrogen bond donor (pyrrole) and acceptor (pyridine) groups, which leads to the formation of cyclic dimers in their crystals. Herein, we present a joint experimental (Raman scattering) and computational (DFT modelling) study on the high-pressure behaviour of PQ and PP molecular crystals. Our results indicate that compression up to 10 GPa (100 kbar) leads to considerable strengthening of the intermolecular hydrogen bond within the cyclic dimers. However, the intramolecular N–H∙∙∙N interaction is either weakly affected by pressure, as witnessed in PQ, or weakened due to compression-induced distortions of the molecule, as was found for PP. Therefore, we propose that the compression of these systems should facilitate double proton transfer within the cyclic dimers of PQ and PP, while intramolecular transfer should either remain unaffected (for PQ) or weakened (for PP).

## 1. Introduction

Proton-transfer (PT) reactions are at the heart of many fundamental chemical and biological processes, such as acid–base neutralization [1], electron transfer [2], and enzymatic reactions [3]. An important class of PT processes includes those where the reaction is initialized by placing the molecule in an excited electronic state—the so-called excited-state proton transfer. When the migration of the proton is confined within one molecule, such a reaction is described as an excited-state intramolecular proton transfer (ESIPT) [4]. The study of systems exhibiting ESIPT receives much attention not only because of its importance in understanding molecular properties and intermolecular interactions [5,6,7,8], but also due to possible technological applications [9].

The appearance of a hydrogen bond is a prerequisite for PT reactions, both inter- and intramolecular, and the characteristics of this bond strongly influence the fate of the proton transfer. In case of systems exhibiting ESIPT, both the hydrogen-bond donor (hydroxyl, amino, or pyrrolic groups) and acceptor (carbonyl oxygen, pyridyl, or azole nitrogen) sites belong to the same molecule and lie not far apart from each other [10]. Among several systems exhibiting such an intramolecular hydrogen bond those containing the pyridine–pyrrole pair are of interest due to the directional nature of the pyrrolic N–H bond [11,12,13,14,15,16,17,18]. In these molecular systems, studied mostly in the gas phase or solution, the geometry of the intramolecular hydrogen bond was altered through chemical modification to the σ-bond backbone. High pressure (exceeding 1 GPa = 10 kbar) offers an alternative route for modifying intra- and intermolecular geometry. High-pressure experiments on molecular crystals yield important information on intermolecular interactions, in particular hydrogen bonds [19,20,21,22,23,24,25,26]. Pressure can be also used to tune the electronic and spectroscopic properties of various systems [27].

**Figure 1:**
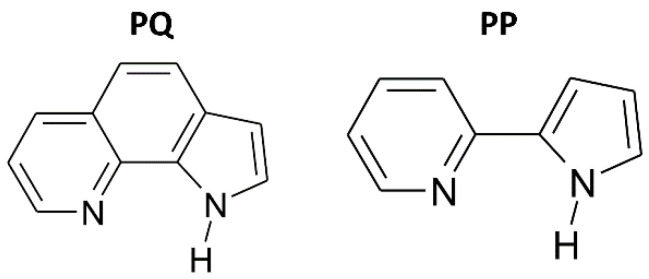
1*H*-pyrrolo[3,2-*h*]quinoline (PQ) and the *syn* conformer of 2-(2′-pyridyl)pyrrole (PP).

Here we present a high-pressure experimental and computational study on molecular crystals of two compounds containing a strong intramolecular N–H∙∙∙N interaction: 1*H*-pyrrolo[3,2-*h*]quinoline (PQ) and 2-(2′-pyridyl)pyrrole (PP)—see Figure 1. Raman spectroscopy, supplemented with solid-state density-functional-theory (DFT) calculations, is used to elucidate the structural behaviour of these systems up to pressures of 10 GPa. In particular, we analyse the pressure-induced changes in the intermolecular hydrogen bond within the cyclic dimers formed by these molecules in the solid state, as well as in the intramolecular N–H∙∙∙N interaction.

## 2. Results

### 2.1. High-Pressure Behaviour of PQ

The pyrrole part of the PQ molecule is linked with the pyridyl part by a stiff vinyl bridge. This ensures correct orientation of both nitrogen atoms, in contrast to PP where the single C-C bridge allows for the rotation of the two parts with respect to each other. Nevertheless, the rigidity of PQ results in geometric restraints on the intramolecular N–H∙∙∙N interaction, which leads to its weakening with respect to PP. As a result a considerable barrier exists for the intramolecular proton-transfer process hindering observation of ESIPT for the PQ molecule [12]. However, double-proton transfer with alcohols and water is observed when PQ forms cyclic dimers with those solvents [12,16,28,29].

We note that, in both PP and PQ, the angle along the intramolecular N-H∙∙∙N interaction (circa 90°) can be viewed as too small to consider it a hydrogen bond. However, presence of a considerable intramolecular interaction between these atoms is supported by the redshift of the N-H stretching mode in isolated molecules: 3530 cm^−1^ in pyrrole [30] compared to 3456 (−74) cm^−1^ in PQ [31], and 3483 (−47) cm^−1^ in PP [32]. Adding to this spectroscopic signature is the difference in the calculated frequency of this vibration in the *anti* (3678 cm^−1^) and *syn* (3637 cm^−1^) conformers of PP. The considerable strength of the N–H∙∙∙N interaction can be linked to the short N∙∙∙N distance in both compounds (2.99 Å in PQ [31], 2.79 Å in PP [33]).

**Figure 2:**
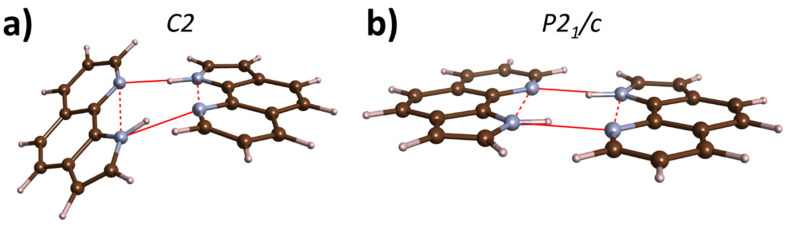
PQ dimers present in the crystal structure of the (**a**) *C2* and (**b**) *P2*_1_*/c* polymorphs. Brown/blue/grey balls mark C/N/H atoms; full/dotted red lines mark N∙∙∙N contacts along the intermolecular hydrogen bond and the N–H∙∙∙N intramolecular interaction.

Two crystal structures have been reported for PQ at ambient conditions—one of *C2* symmetry (*Z* = 8) [34], the other with *P2*_1_*/c* symmetry (*Z* = 4) [31]. Both contain hydrogen-bonded cyclic dimers of PQ molecules, which are bent for the *C2* polymorph and planar for *P2*_1_*/c* (Figure 2).

**Figure 3:**
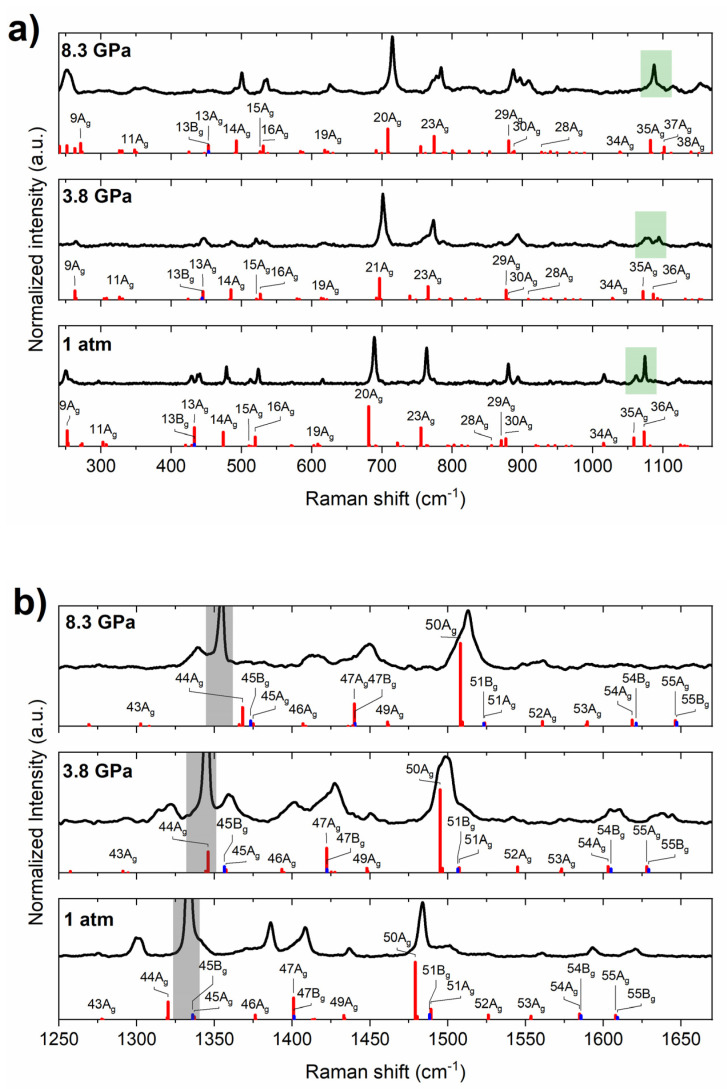
Experimental Raman spectrum of solid PQ at selected pressures (black lines) in the range of Raman shifts from (**a**) 200–1200 cm^−1^ and (**b**) 1200–1700 cm^−1^, together with Raman intensities simulated with the PBE+TS method for the *P2*_1_*/c* structure (red/blue bars for modes of A_g_/B_g_ symmetry). The labels of the most intense Raman modes are given. Green boxes in (**a**) indicate the region of the skeletal deformation bands for which a change of intensity is observed upon compression. Grey boxes in (**b**) mark the first-order Raman signal from the diamond anvil.

The ambient-pressure spectra of the two polymorphs are similar with the largest difference found for two relatively intense peaks located at 1062 and 1074 cm^−1^ [31]. For the *P2*_1_*/c* structure the lower energy peak is less intense, while the opposite is found for the *C2* polymorph. The ambient-pressure Raman spectrum of PQ used in our study is consistent with that of the *P2*_1_*/c* structure (Figure 3), confirming that the high-pressure measurements start from this polymorph.

Upon compression of powdered PQ, the colour of the sample changes from off-white at 1 atm to light orange (see Figure S1 in the Supplementary Materials) indicating a reduction of the band gap. This process leads to an increase in the fluorescence background, which prevents the observation of Raman bands above 10 GPa. In order to acquire information on the possible high-pressure phase transition of PQ, we analysed the Raman spectrum of this compound from 1 atm to 10 GPa, focusing on the region of molecular vibrations from 200 to 1700 cm^−1^, where about 30 bands could be observed. Higher-frequency molecular bands (C-H and N-H stretches found above 3000 cm^−1^) could not be detected at large compression due to low intensity and large background. Lattice modes resulting from intermolecular vibrations, found at Raman shifts lower than 200 cm^−1^, were also omitted from the analysis due to large peak overlap.

Previous Raman scattering measurements for the *P2*_1_*/c* polymorph conducted up to 3.5 GPa showed that the most prominent change in the Raman spectrum observed upon compression was the exchange of intensities of two bands located at 1062 and 1074 cm^−1^ at ambient pressure [31]. In other words, at high pressure, the intensity pattern of these two peaks resembles that found at ambient pressure for the *C2* polymorph. Based on this it was suggested that compression leads to the formation of twisted dimers resembling those found in the *C2* structure.

Our Raman measurements, extended to 10 GPa, reproduce the intensity switch of the 1062 and 1074 cm^−1^ peaks. The lower energy band has smaller intensity at 1 atm, but as pressure increases, the intensity of this band becomes higher at the expense of the high-energy band. At approximately 4 GPa, the bands are of the same intensity, while at 8 GPa, the lower-energy one has higher intensity (Figure 3). These changes are reversible upon the decompression of the sample.

In order to gain more information on the behaviour of the *P2*_1_*/c* polymorph of PQ at large compression, we simulated the vibrational properties of this structure using the DFT method. Previous studies indicated that the PBE functional, one of the most popular generalized-gradient approximation (GGA) functionals, offers a reasonable description of the frequencies and intensities of Raman active vibrations of the PQ molecule [35]. Thanks to the low computational cost of PBE, as compared to meta-GGA or hybrid functionals [36], we were able to calculate not only the frequencies of Γ-point vibrational modes but also their Raman activity, as well as the phonon dispersion. We note that van der Waals interactions, poorly modelled with GGA functionals, were accounted for with the use of the dispersion correction of Tkatchenko and Scheffler [37] (for more details, see Materials and Methods).

**Figure 4:**
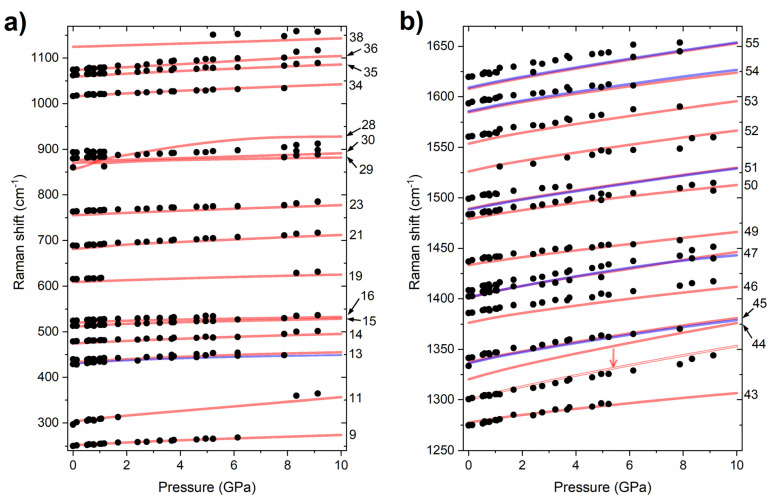
Pressure dependence of the experimental frequencies of Raman bands of compressed PQ in the range of Raman shifts from (**a**) 200–1200 cm^−1^ and (**b**) 1200–1700 cm^−1^ (black dots). Red/blue lines mark the simulated frequencies for the most intense modes of A_g_/B_g_ symmetry (mode numbers are given). The frequencies, obtained with the PBE functional, were not scaled—the result of scaling down of the 44 A_g_ mode by 1.5% is shown by an arrow and double line.

The PQ molecule is characterized by 57 molecular vibrational modes; therefore, the *P2*_1_*/c* structure, which contains 4 molecules per formula unit, exhibits 228 molecular vibrations and 24 intermolecular (lattice) modes, totalling 252 modes. Group theory analysis indicates that, for the centrosymmetric *P2*_1_*/c* structure, only half of these will be Raman-active with 63 modes of A_g_ symmetry, and 63 of B_g_ symmetry. As can be seen in Figure 3, computations indicate that the most intense Raman bands are those of A_g_ symmetry. The intensities derived from theory are in good accordance with those obtained experimentally, both at ambient and high pressure. Most importantly, the difference in intensities of the 1062 and 1074 cm^−1^ bands between the *P2*_1_*/c* and *C2* polymorphs at 1 atm is reproduced in calculations (Figure S2 in the Supplementary Materials). Moreover, the observed intensity change of these bands upon compression is reproduced under the assumption that PQ remains in the *P2*_1_*/c* structure (Figure 3). This suggests that no phase transition is observed throughout the experiment (up to 10 GPa). This result is further corroborated by the excellent agreement between the experimental Raman frequencies and those modelled for the *P2*_1_*/c* structure (Figure 4). The only major disagreement is found for the 44A_g_ mode for which theory overestimates the frequency by 25 cm^−1^ (ca. 1.5%). Inspection of the eigenvector of this mode reveals that it involves considerable movement of the N-bonded hydrogen in the direction perpendicular to the N-H bond. Therefore, the overestimation of the frequency of this vibration most likely stems from large anharmonic effects associated with the hydrogen bond.

**Figure 5:**
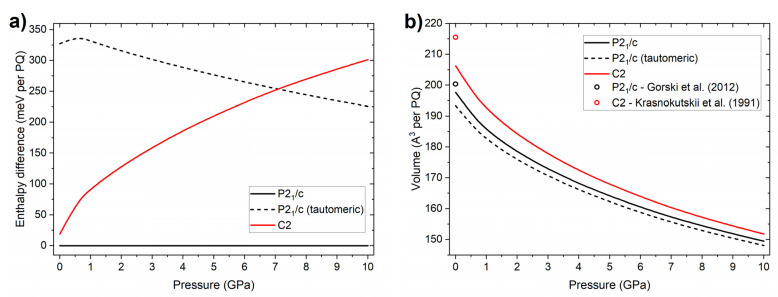
Pressure dependence of the (**a**) relative enthalpy (referenced to that of the *P2*_1_*/c* structure) and (**b**) volume of three PQ polymorphs: *P2*_1_*/c*, *C2*, and the tautomeric variant of *P2*_1_*/c*. Lines mark results of PBE+TS calculations; dots mark experimental volumes at 1 atm.

In order to confirm the lack of a phase transition for compressed PQ, we performed DFT geometry optimization and enthalpy calculations for the *P2*_1_*/c* and *C2* structures of this compound up to 10 GPa. Comparing the enthalpies of these polymorphs, we find them to be energetically nearly degenerate at 1 atm (effectively 0 GPa). At this pressure, the *C2* structure is higher in energy compared to *P2*_1_*/c* by 19 meV per PQ molecule (16.5 meV after including zero-point energy vibrations). This small difference, which corresponds to a temperature of 220 K, explains why the metastable *C2* polymorph can be observed experimentally. However, compression destabilizes this structure with respect to *P2*_1_*/c*, as shown in Figure 5a. At 10 GPa, the enthalpy difference between those structures reaches 301 meV (6.9 kcal/mol). The reason behind this behaviour is the larger volume of the *C2* structure compared to *P2*_1_*/c* (Figure 5b)—in other words, better molecule-packing favours the latter structure at high pressure.

The fact that the *C2* polymorph is metastable with respect to *P2*_1_*/c* at ambient and high pressure clearly shows that a *P2*_1_*/c*→*C2* phase transition is not preferred thermodynamically and corroborates the experimental observation of the lack of a phase transition upon compression of the *P2*_1_*/c* polymorph. We also verified that the *P2*_1_*/c* structure is vibrationally (dynamically) stable up to 10 GPa with no imaginary modes present in its phonon structure (Figure S3 in the Supplementary Materials).

**Figure 6:**
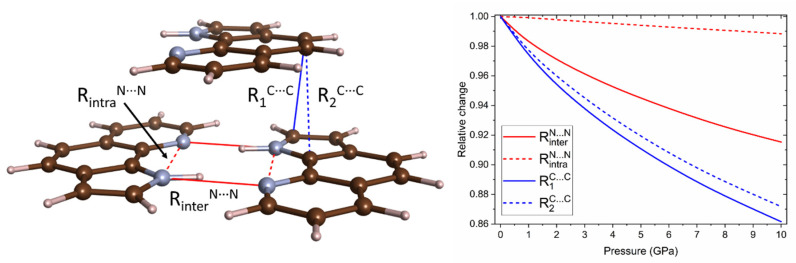
Pressure dependence of the distance of closest N∙∙∙N and C∙∙∙C contacts in the *P2*_1_*/c* structure of PQ simulated with the PBE+TS method.

Analysis of the DFT-derived geometry of the *P2*_1_*/c* polymorph of PQ at various pressures shows that the molecule itself, as well as the cyclic dimers, remains planar upon compression. Pressure has little influence on the intramolecular N–H∙∙∙N interaction (Figure 6 and Table 1), as the intramolecular N∙∙∙N distance contracts only by 1.2% upon compression to 10 GPa. This is a result of the stiffness of the aromatic backbone of PQ molecules. In contrast, intermolecular hydrogen bonds are considerably strengthened upon compression, as witnessed by an 8.5% decrease in the intermolecular N∙∙∙N distance, connected with a 1.5% elongation of the N-H bond (this can be compared with a 0.7% shortening of C-H bonds in the same pressure range). We note that a more detailed analysis of the interactions involved in the studied hydrogen bonds (as described in [38]), although of interest, is beyond the scope of the current study.

Interestingly, we find that C∙∙∙C contacts between neighbouring PQ molecules are the ones most affected by compression, with a maximum 14% contraction from 1 atm to 10 GPa. This leads to an increase in the stacking interaction between pairs of molecules that are not hydrogen-bonded. A similar increase in stacking interactions was observed in a number of systems [39,40,41]. Most notably, in the case of pyrrole, compression leads to formation of a crystal structure consisting of dimers exhibiting short C∙∙∙C contacts [41]. This high-pressure polymorph is a precursor for the pressure-induced polymerization of pyrrole. We speculate that a similar mechanism might be applicable in the case of the possible polymerization of PQ at pressures exceeding 10 GPa.

**Figure 7:**
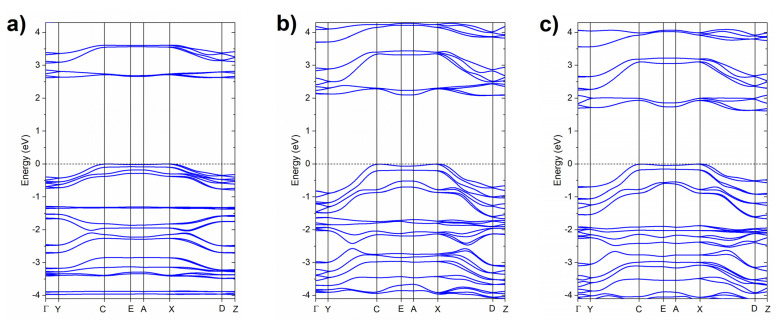
Electronic band structure (in the vicinity of the Fermi level) simulated with the PBE functional for PQ in the *P2*_1_*/c* structure at (**a**) 1 atm and (**b**) 10 GPa and for (**c**) the tautomeric variant of *P2*_1_*/c* at 10 GPa. In all graphs, the energy of the top of the valence band is set to 0 eV.

Electronic-band-structure DFT calculations indicate a decrease of the band gap by 0.54 eV up to 10 GPa (Figure 7), in agreement with the experimentally observed change of the colour of PQ from white to orange. We note that the *P2*_1_*/c* structure of PQ exhibits an indirect band gap with the top of the valence band at C (0, ½, ½) and X (0, ½, 0) points of the Brillouin zone, while the bottom of the conduction band at E (½, ½, ½) and A (½, ½, 0) k-vectors. The energy difference between the valence and conduction bands at these points is equal to 2.61 eV at 1 atm and 2.07 eV at 10 GPa. The band gap at Γ-point is larger, and almost insensitive to pressure (3.01 at 1 atm and 2.95 eV at 10 GPa). The ambient-pressure value of the Γ-point band gap is in fair agreement with the experimental singlet excitation energy (3.66 eV) found for the isolated molecule [28].

The enhancement of the intermolecular hydrogen bond in PQ crystals might facilitate intermolecular double proton transfer between the cyclic dimers, in analogy to what is found for complexes of PQ with alcohols [12]. On the other hand, intramolecular proton transfer, which is kinetically hindered for the isolated molecule [16], should remain inactive at high pressure. To further explore the energetics of proton exchange in the *P2*_1_*/c* structure of PQ, we performed calculations for a tautomeric variant of this crystal, in which both pyrrole hydrogen atoms in the cyclic dimer were migrated to the pyridine fragment, and the whole geometry of the crystal was optimized (see Figure S4). At ambient pressure, this tautomer has an energy 325 meV (7.5 kcal/mol) higher than the normal *P2*_1_*/c* structure. This difference is smaller than calculated for the isolated molecule (21.0 kcal/mol) [16] and closer to that calculated for a doubly hydrogen-bonded complex of PQ and two water molecules (12.8 kcal/mol), pointing to the stabilizing effect of the formation of cyclic hydrogen bonds between two PQ molecules, although one must keep in mind that both numbers were derived with different DFT functionals.

The enthalpy difference between normal and tautomeric PQ crystals decreases to 230 meV (5.3 kcal/mol) upon compression to 10 GPa (Figure 5a). The stabilization of the tautomeric form stems from its the smaller volume (Figure 5b). The denser packing of the PQ molecules leads to a stronger hydrogen bond forming between tautomeric PQ molecules, as evidenced by a 3.7% reduction in the intermolecular N∙∙∙N distance compared to the normal form (Table 1). As can be seen in Figure 7, the tautomeric form has a band gap of 1.61 eV (2.39 eV gap at Γ-point) at 10 GPa, which is 0.46 eV smaller than that of normal PQ at the same pressure.

### 2.2. High-Pressure Behaviour of PP

The PP molecule can exist in two geometries: the *syn* conformation exhibiting an intramolecular N–H∙∙∙N interaction and the *anti* conformation, in which the rotation of the pyrrole and pyridine rings results in the nitrogen atoms lying on the opposite sides of the carbon bridge. The *syn* conformation is the ground state in the gas phase [42,43]; it is also the dominant form in aprotic solvents [13]. Importantly, the *syn* conformer of PP exhibits the ESIPT process both when solvated and in the gas phase [17,42].

**Figure 8:**
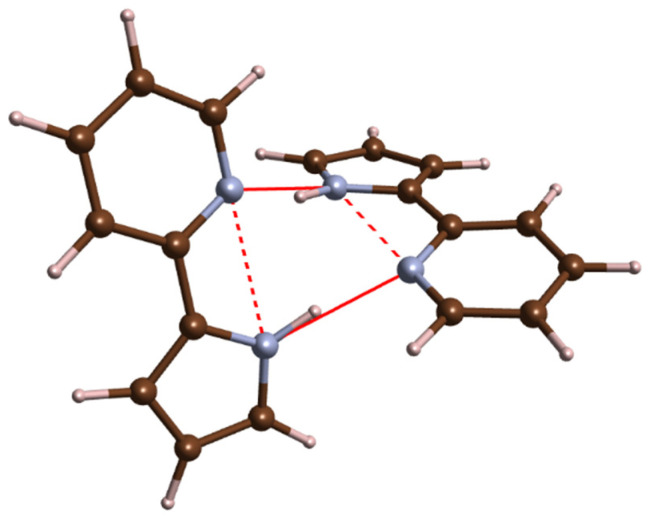
PP dimers present in the crystal structure of *P4*_3_*2*_1_*2* polymorph. Brown/blue/grey balls mark C/N/H atoms; full/dotted red lines mark N∙∙∙N contacts along the intermolecular hydrogen bond and the N–H∙∙∙N intramolecular interaction.

**Figure 9:**
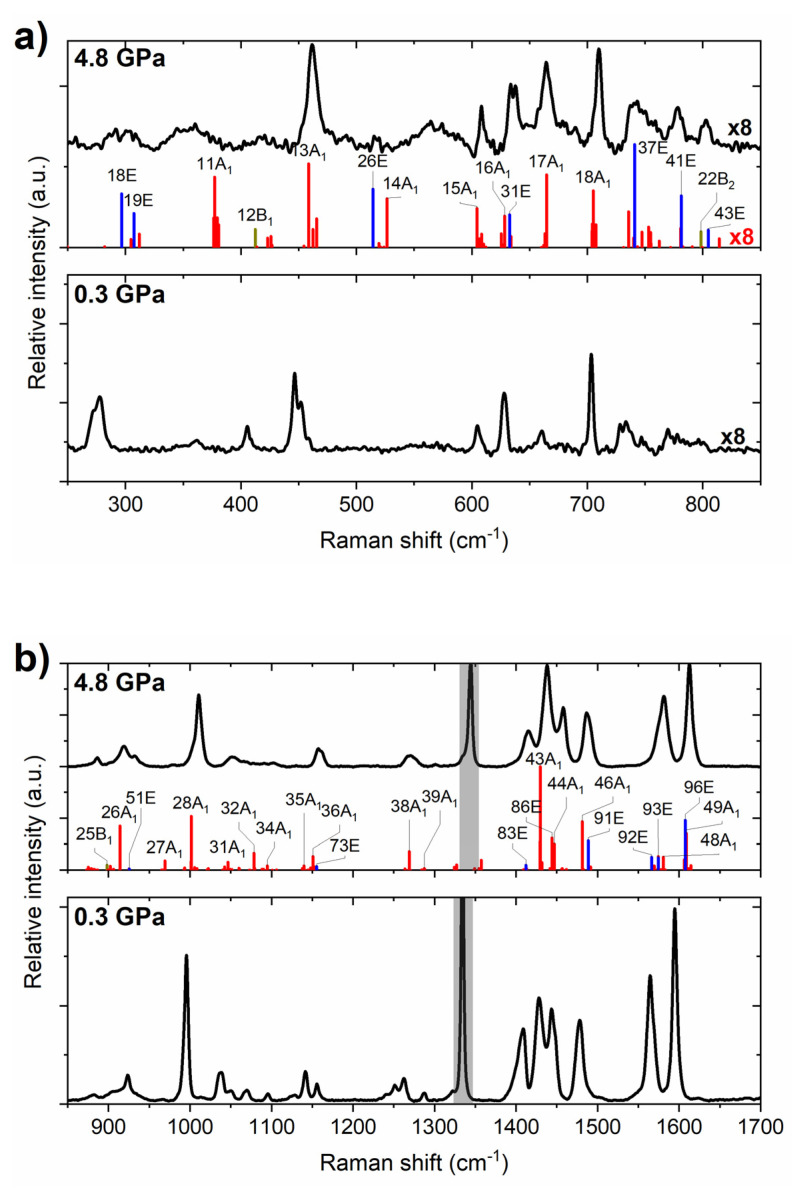
Experimental Raman spectrum of solid PP at 0.3 and 4.8 GPa (black lines) in the range of Raman shifts from (**a**) 250–850 cm^−1^ and (**b**) 850–1700 cm^−1^, together with Raman intensities simulated with the PBE+TS method for the *P4*_3_*2*_1_*2* structure (red/blue/yellow/green bars for modes of A_1_/E/B_1_/B_2_ symmetry). The labels of the most intense Raman modes are given. For clarity, the intensities in the 250–850 cm^−1^ region were multiplied by 8. Grey boxes in (**b**) mark the first-order Raman signal from the diamond anvil.

At ambient conditions, PP forms a crystal with *P4*_3_*2*_1_*2* symmetry (*Z* = 8), exhibiting doubly hydrogen-bonded dimers of PP molecules in the *syn* conformation (Figure 8) [33]. In contrast to the *P2*_1_*/c* structure of PQ, these cyclic dimers are not planar—two PP molecules are twisted with respect to each other with a dihedral angle of 55.6°. Previous vibrational-spectroscopy measurements for solid PP included only an IR absorption experiment for a KBr pellet [32]. We were able to acquire Raman spectrum of this compound in the 200–1700 cm^−1^ range at ambient conditions and pressures up to 7 GPa. As can be seen in Figure 9, pressure does not induce any major changes in the spectra.

**Figure 10:**
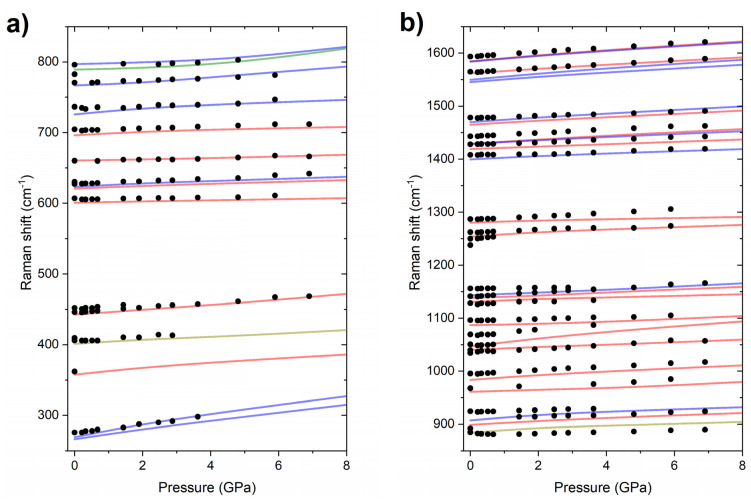
Pressure dependence of the experimental frequencies of Raman bands of compressed PP in the range of Raman shifts from (**a**) 250–850 cm^−1^ and (**b**) 850–1700 cm^−1^ (black dots). Red/blue/yellow/green lines mark the simulated frequencies for the most intense modes of A_1_/E/B_1_/B_2_. The frequencies, obtained with the PBE+TS method, were not scaled.

Using the same computational methodology as for PQ, we have simulated the frequencies and Raman intensities for the vibrational modes for the *P4*_3_*2*_1_*2* structure. The PP molecule exhibits 51 vibrational modes, which means that the crystal will exhibit a total of 456 modes, of which 401 are internal vibrations of the molecules. These modes can be divided according to symmetry into: A_1_, A_2_, B_1_, B_2_ (57 modes in each group) and doubly degenerate E modes (114 modes). Of these, only A_2_ modes are not Raman-active. Given the large computational burden of evaluating the Raman intensities of nearly 400 modes, we performed such calculations only at one selected point (4.8 GPa). As can be seen in Figure 9, there is a good accordance between the spectra simulated assuming the *P4*_3_*2*_1_*2* structure, and the experimental one. We additionally performed calculations of vibrational-mode frequencies in the pressure range up to 8 GPa. This yielded a pressure dependence of the frequency of the most intense Raman bands in good accordance with experiments (Figure 10). These results clearly indicate that, upon compression to 8 GPa, solid PP remains in the *P4*_3_*2*_1_*2* structure. This result is further corroborated by the calculated dynamical stability of this polymorph at 8 GPa (Figure S2 in the Supplementary Materials).

**Figure 11:**
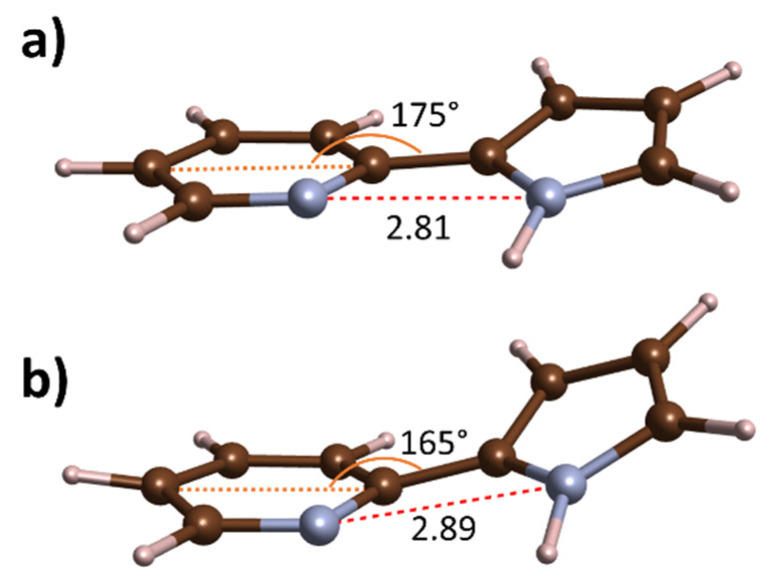
Geometry of a single molecule of PP in the *P4*_3_*2*_1_*2* crystal at (**a**) 1 atm and (**b**) 10 GPa. N∙∙∙N distances along the intramolecular N–H∙∙∙N interaction are given in Å.

Compared to PQ, the PP molecule exhibits a much less rigid connection between the hydrogen donor and acceptor centres. As a result, distortions along some of the PP vibrational modes lower the energy barrier for intramolecular proton transfer enabling observation of ESIPT for this molecule [17,42,43]. In order to examine the influence of compression on the PP molecule conformation in the *P4*_3_*2*_1_*2* structure, we performed DFT geometry optimization for this polymorph at various pressures. At 1 atm, the PP molecule in this crystal is nearly planar with a small rotation of the pyridine and pyrrole units along the C-C bridge (dihedral angle of 10.6°)—in accordance with the experimental structure [33]. The pyrrole ring is additionally slightly elevated above the plane formed by the pyridine ring, as shown in Figure 11a.

One might expect that, upon compression, the nitrogen centres of both rings will be brought together, resulting in the strengthening of the intramolecular N–H∙∙∙N interaction. Our simulations indicate that the opposite happens—the intramolecular N∙∙∙N distance becomes larger by 2.9% upon compression to 10 GPa. This is a result of substantial distortion induced on the molecule—most notably the greater departure of the pyrrole ring from the plane formed by the pyridine fragment (Figure 11b). This indicates that compression weakens the intramolecular N–H∙∙∙N interaction. At the same time, the intermolecular hydrogen bond is strengthened as witnessed by a shortening of the intermolecular N∙∙∙N contacts from 2.87 Å at 1 atm to 2.66 Å at 10 GPa (7.2% decrease). This is accompanied by the lengthening of the N-H bonds by 1.2% (from 1.05 to 1.06 Å).

DFT calculations indicate that the influence of pressure on the band gap of the *P4*_3_*2*_1_*2* crystal of PP is similar as found for PQ. In ambient conditions, *P4*_3_*2*_1_*2* exhibits a direct band gap (at M point—½, ½, 0) of 2.83 eV. The band gap at Γ-point is almost identical. Upon compression, the band gap decreases to 2.48 eV (also at M point) with the gap at Γ-point only slightly larger (2.58 eV).

## 3. Discussion

Our combined experimental and theoretical study on the high-pressure behaviour of molecular crystals of PQ and PP indicates the absence of any phase transition in both systems up to 10 GPa and 8 GPa, respectively. We found that, of the two PQ polymorphs, the one containing planar cyclic dimers (*P2*_1_*/c*) is the ground state at ambient and high pressure, and that compression does not induce the bending of the dimers. Similarly, the cyclic dimers found in the crystal of PP are retained upon compression, although the molecule itself experiences large distortions. This leads to a lengthening of the intramolecular N∙∙∙N contacts and therefore, should result in the weakening of the intramolecular N–H∙∙∙N interaction. In case of PQ, this interaction is only slightly strengthened upon compression. Despite the large differences in the geometry of the hydrogen-bonded dimers found in PQ and PP crystals, pressure affects intermolecular hydrogen bonds similarly in both systems. Their considerable shortening is witnessed upon compression.

The above results hint that high pressure should facilitate double proton transfer within cyclic PP and PQ dimers, while ESIPT should either remain unaffected (for PQ) or weakened (for PP). DFT calculations indicate a reduction of the band gap with pressure, which suggests a redshift of the absorption peaks upon compression. These results will hopefully serve as a basis for future studies on the electronic and excited-state properties of these systems at large compression.

## 4. Materials and Methods

Synthesis: The synthesis and purification of 1*H*-pyrrolo[3,2-*h*]quinoline (PQ) was based on a previously published procedure [44], although with several modifications, in particular concerning the purification process. The synthesis of 2-(2′-pyridyl)pyrrole (PP) was conducted through the coupling of 1-(*tert*-butoxycarbonyl)pyrrole-2-yl)boronic acid (synthesised as described in ref. [45]) with 2-bromopyridine. Details of the synthetic procedures are given in the Supplementary Materials.

High-pressure experiments: High-pressure measurements were conducted with the use of a diamond anvil cell (DAC [46]) equipped with IIas diamonds with a 400 µm (PP) and 500 µm (PQ) culet. The sample was enclosed between the culets of the opposing diamonds and a stainless-steel gasket pre-indented to a thickness of circa 30 µm. The gasket hole (radius of 180 µm) was laser-drilled. The sample space, containing several ruby chips, was filled with powdered samples of both compounds after which the DAC was closed. No pressure-transmitting medium was used. The pressure was determined with the use of the ruby fluorescence scale (IPPS-Ruby2020) [47].

Raman spectroscopy: Raman spectra were acquired with the Alpha300M+ confocal microscope (WITec Gmbh) equipped with a motorised stage. The confocal set-up enabled acquiring the signal from a small portion of the sample (approximately 2 × 2 × 6 µm^3^) located only a few µm from the ruby used for pressure determination. This partly alleviated the problems associated with pressure gradients inside the DAC that result from the lack of a pressure-transmitting medium. The good accordance between experimental Raman shifts and those simulated for hydrostatic conditions (see Figure 4 and Figure 10) indicated that the probed sample region exhibited quasi-hydrostatic conditions.

We used 633 nm (PQ) and 532 nm (PP) laser lines delivered to the microscope through single-mode optical fibers. The laser power at the sample did not exceed 10 mW for PQ and 5 mW for PP. The backscattered Raman signal was collected through a 20× long working distance objective, and passed through a photonic optical fibre to a lens-based spectrometer (Witec UHTS 300, f/4 aperture, focal length 300 mm) coupled with a back-illuminated Andor iDUS 401 detector thermoelectrically cooled to −60 °C. The spectra were collected in the range of Raman shifts from 100 to 1700 cm^−1^ with the use of an 1800 mm grating. Due to the poor signal-to-intensity ratio, we were not able to analyse the bands appearing in the range of C-H/N-H stretching vibrations (above 3000 cm^−1^). Typical acquisition time was 1 s with 30 accumulations. The spectra were post-processed (background subtraction and cosmic-ray removal) with Project FIVE software (Witec Gmbh), and normalized to the intensity of the strongest band of PQ (1483 cm^−1^ at 1 atm) and PP (1595 cm^−1^ at 1 atm). The position of Raman bands was established with Fityk 1.3.1 software by fitting the observed bands with pseudo-Voigt profiles [48]. At selected pressures, Raman mapping of the whole sample enclosed in the DAC was conducted in order to confirm th sample integrity.

DFT calculations: Periodic density functional theory (DFT) calculations of the geometry, electronic structure, and enthalpy of PQ and PP crystals at ambient and elevated pressure utilized the PBE functional [49], as implemented in CASTEP (version 19.11) [50]. In order to properly account for van der Waals interactions between molecules in the crystal lattice, we used the dispersion correction of Tkatchenko and Scheffler (TS correction) [37]. We found that the chosen method, which was successfully applied in a recent study of the high-pressure phase transitions of chloroform and dichloromethane [25], reproduces very well the geometry of the known structures of both compounds with differences not exceeding 4% (see Tables S1 and S2 in the Supplementary Materials). Thermodynamic stability of PQ polymorphs was judged by comparing their enthalpy (*H*), and thus, the calculations formally correspond to *T* = 0 K, at which point the Gibbs free energy (*G* = *H* − *S × T*, where *S* is the entropy) is equal to the enthalpy. Zero-point energy (ZPE) corrections were only included in the calculations conducted at 1 atm for the *P2*_1_/c and *C2* structures of PQ.

The valence electrons were described with a plane-wave basis set (1100 eV cut-off), while norm-conserving pseudopotentials were used for the description of core electrons. The convergence criterion for the electronic minimization was 10^−7^ eV per atom. Sampling of the Brillouin zone was done through a Monkhorst–Pack mesh [51] with a 2π × 0.05 Å^−1^ spacing of *k*-points. The geometry optimization of the crystal structures was performed with the use of the Broyden–Fletcher–Goldfarb–Shanno scheme [52]. Structures were optimized until the forces acting on the atoms were smaller than 5 meV/Å, the difference between the applied hydrostatic pressure and all of the stress components was smaller than 0.05 GPa, and the maximum ionic displacement was smaller than 5 × 10^−4^ Å.

We also used CASTEP for calculating the frequency and intensity of Γ-point Raman-active vibrational modes using density-functional perturbation theory (DFPT) [53]. The Raman activity of each vibrational mode (*S_i_*) was converted into the intensity (*I_i_*) assuming the following relation:

Ii ~ (ν0−νi)4νi(1−e−hνickT)Si,

where *ν*_0_ is the laser frequency; *ν_i_* is the mode frequency, and *T* is the temperature (taken as equal to 293 K). Visualization of all structures was performed with the VESTA software package [54].

## Figures and Tables

**Table 1:** Comparison of the calculated geometry of PQ in the *P2*_1_*/c* structure at 1 atm and 10 GPa (labelling follows that of Figure 6), as well as the tautomeric variant of PQ at 10 GPa. Distances are given in Å.

Contact	*P2*_1_ */c*		*P2*_1_*/c* (Tautomeric)
	1 atm	10 GPa	10 GPa
$R_{inter}^{N\cdots N}$	2.96	2.71	2.61
$R_{intra}^{N\cdots N}$	3.02	2.98	3.01
$R_{1}^{C\cdots C}$	3.35	2.89	2.89
$R_{2}^{C\cdots C}$	3.30	2.88	2.88
$R_{N-H}$	1.04	1.05	1.12

## Data Availability

Data available on request from the corresponding author.

## Supplementary Materials

The following are available online, Tables S1 and S2: Comparison of the calculated and experimental geometry of the PP and PQ structures known at ambient conditions; Figure S1: Bright-field image of powdered PQ enclosed in a DAC at 0.6 and 11.0 GPa; Figure S2: Raman spectrum of PQ crystals at 1 atm together with the spectra simulated for the *P2*_1_*/c* and *C2* polymorph; Figure S3: Calculated phonon dispersion for the *P2*_1_*/c* structure of PQ at 10 GPa, and the *P4*_3_*2*_1_*2* polymorph of PP at 8 GPa; Figure S4: Dimers present in the crystal structure of the tautomeric form of the *P2*_1_*/c* polymorph; details of synthesis and purification of PP and PQ.

## Sample Availability

Samples of the compounds 1*H*-pyrrolo[3,2-*h*]Quinoline (PQ) and 2-(2′-Pyridyl)pyrrole (PP) are available from the authors.
