# Supplementary material for: Hydrogen-Bonded Cyclic Dimers at Large Compression: The Case of 1H-pyrrolo[3,2-h]quinoline and 2-(2′-pyridyl)pyrrole
Source: Molecules. 2021 Jun 22;26(13):3802. doi: 10.3390/molecules26133802 (PMC8270273; doi:10.3390/molecules26133802)
Supplement: Supplementary file 1 [file molecules-26-03802-s001.zip › molecules-1256533-supplementary.pdf]

molecules

SUPPLEMENTARY MATERIALS

Hydrogen-bonded cyclic dimers at large compression: case of 1*H*-pyrrolo[3,2-*h*]quinoline and 2-(2′-pyridyl)pyrrole

Table S 1 Comparison of the calculated and experimental geometry of the two PQ structures known at ambient conditions. Cell vector length and atomic distances are given in Å, angles in degrees, volume in Å^3. Percentage difference between experimental data and calculations is given in parenthesis.

PQ ($P2_1/c$) p = 1 atm	Exp. (ref. [1])	PBE+TS (this work)		PQ ($C2$) p = 1 atm	Exp. (ref. [2])	PBE+TS (this work)	
**a**	9.010	9.002	(–0.1 %)	**a**	25.518	25.495	(–0.1 %)
**b**	4.730	4.656	(–1.6 %)	**b**	6.257	6.036	(–3.5 %)
**c**	19.312	19.393	(+0.4 %)	**c**	12.085	11.994	(–0.8 %)
**β**	103.18	103.42	(+0.2 %)	**β**	116.67	116.36	(–0.3 %)
**V**	801.39	790.66	(–1.3 %)	**V**	1724.27	1653.63	(–4.1 %)
**R(N···N)$_{intra}$**	2.993	3.020	(+0.9 %)	**R(N···N)$_{intra}$**	2.944	3.012	(+2.3 %)
					2.991	3.009	(+0.6 %)
**R(N···N)$_{inter}$**	3.013	2.964	(–1.6 %)	**R(N···N)$_{inter}$**	2.916	2.894	(–0.8 %)
					2.991	2.882	(–3.6 %)

Table S 2 Comparison of the calculated and experimental geometry of the ambient pressure structure of PP. Cell vector length and atomic distances are given in Å, angles in degrees, volume in Å^3. Percentage difference between experimental data and calculations is given in parenthesis.

PP ($P4_32_12$) p = 1 atm	Exp. (ref. [3])	PBE+TS (this work)	
**a**	8.123	7.882	(–2.9 %)
**c**	23.502	24.229	(+3.1 %)
**V**	1550.74	1505.45	(–2.9 %)
**R(N···N)$_{intra}$**	2.786	2.809	(+0.8 %)
**R(N···N)$_{inter}$**	2.949	2.870	(–2.7 %)

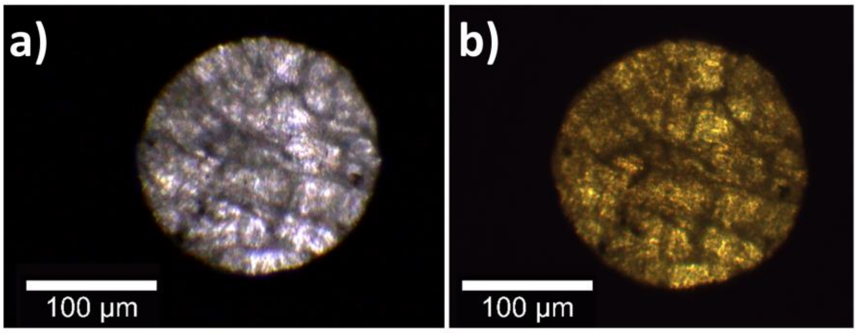

Figure S 1 Bright-field image of powdered PQ enclosed in a DAC at (**a**) 0.6 and (**b**) 11.0 GPa.

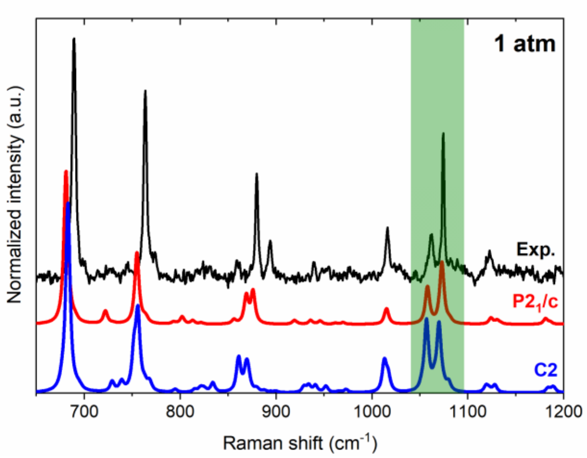

Figure S 2 Raman spectrum, in the 650 – 1200 cm^{-1} range, of PQ crystals at 1 atm (black line), together with the spectra simulated for the *P2$_1$/c* (red line) and *C2* (blue line) polymorphs. The theoretical spectrum was obtained by applying to each Raman peak a Lorentzian broadening with a FHWM of 5 cm^{-1}. Raman bands whose intensity allows for the distinction between the *P2$_1$/c* and *C2* polymorphs are highlighted in green.

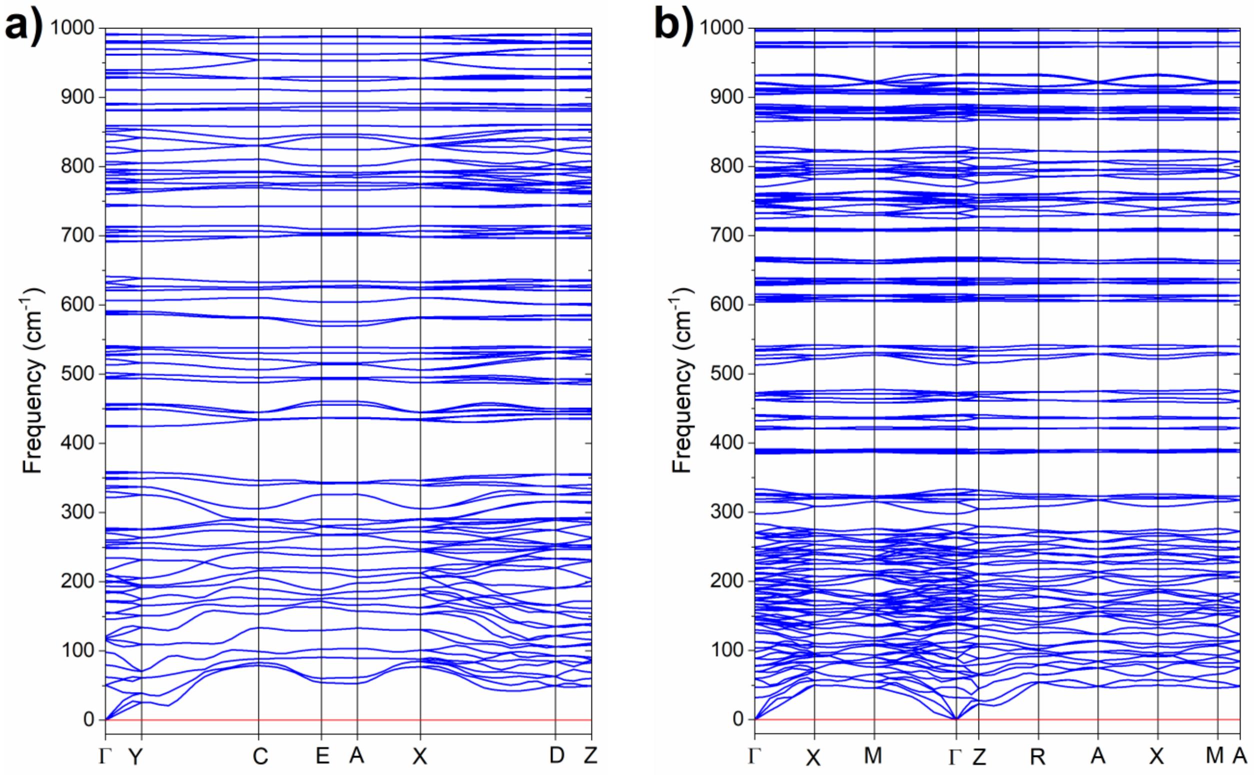

Figure S 3 Calculated phonon dispersion (in the 0 – 1000 cm–1 range) for the (a) P21/c structure of PQ at 10 GPa, and (a) (b) the P43212 polymorph of PP at 8 GPa.

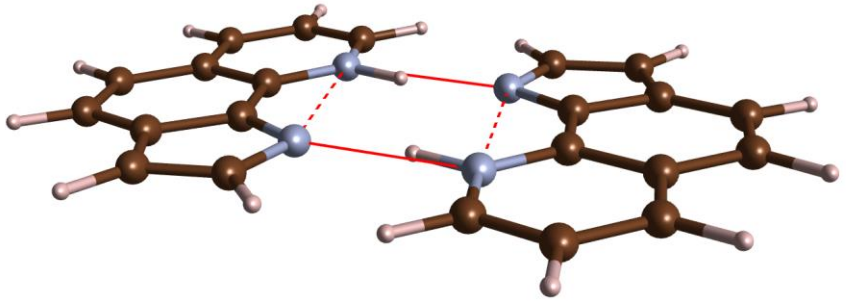

Figure S 4 Dimers present in the crystal structure of the tautomeric form of the *P2*$_1$*/c* polymorph. Brown/blue/grey balls mark C/N/H atoms; dotted/full red lines mark N···N contacts along the intramolecular/intermolecular hydrogen bond.

Synthesis and purification of 1H-Pyrrolo[3,2-h]quinoline (PQ)

<u>7-Methyl-8-nitroquinoline</u>

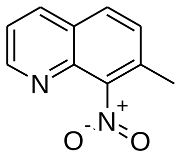

The reaction was carried out under anhydrous conditions. *t*-BuOK (11.32 g, 0.1012 mol) was suspended in THF (40 mL) and the mixture was cooled to 50 °C under vigorous stirring. A solution of 8-nitroquinoline (8.0 g, 0.046 mol) and ethyl chloroacetate (5.35 mL, 0.0504 mol) in THF (40 mL) was added dropwise to maintain the temperature in the range of -55 °C to -40 °C. Then it was allowed to warm up to 0 °C. After 2h water (80 mL) was added and the mixture was stirred at room temperature for additional 4h. Then it was acidified to pH=1 using conc. chloric acid. The mixture was stirred at 70 °C overnight. After cooling it to r.t. pH was rinsed to 4 with a water solution of NaOH. The precipitated solid was collected, washed with hot ethyl acetate (5×200 mL) and discarded. The combined organic solutions were filtered and concentrated to give 7-methyl-8-nitroquinoline (2.63 g, 0.014 mol, 30%) as a pink, crystalline solid.

<u>(1*E*)-*N*,*N*-Dimethyl-2-(8-nitro-7-quinolinyl)ethenamine</u>

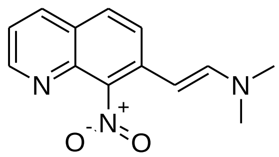

The reaction was carried out under anhydrous conditions. 7-methyl-8-nitroquinoline (1.20 g, 6.35 mmol) was dissolved under argon in anhydrous DMF (2.15 mL), followed by addition of DMFDMA (1.71 mL). A flask with the reaction mixture was placed in the oil bath, heated to 140-150 °C and the reaction mixture was stirred for 12h. Then it was cooled to room temperature, concentrated, and diethyl ether (5 mL) was added to the oily residue. The precipitated solid was washed twice with ether and it was dried on air, to give (1*E*)-*N*,*N*-Dimethyl-2-(8-nitro-7-quinolinyl)ethenamine (0.856 g, 3.52 mmol, 55%) as a bright red, crystalline solid.

molecules MDPI

1*H*-Pyrrolo[3,2-*h*]quinoline

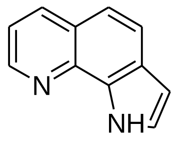

(1*E*)-*N*,*N*-Dimethyl-2-(8-nitro-7-quinolinyl)ethenamine (0.507 g, 2.08 mmol) was dissolved in a mixture of CH_2Cl_2 (16 mL), MeOH (65 mL) and acetic acid (4.3 mL) and Pd/C (10%, 0.226 g) was added. The reaction flask was filled with hydrogen and the reaction mixture was stirred overnight at r.t. Then it was filtered through a Celite pad, which was additionally washed with methanol. The filtrate was concentrated, treated with 5% water solution of $NaHCO_3$ and then extracted twice with ethyl acetate and then with CH_2Cl_2. The combined organic layers were concentrated and purified on column chromatography (hexanes/ethyl acetate; 4/1 then 3/2). The more polar product was collected as an oily, brown liquid, which solidified after three days. The pure 1*H*-Pyrrolo[3,2-*h*]quinoline (0.071 g, 0.422 mmol) was obtained after sublimation in 20% yield.

Synthesis and purification of 2-(2'-pyridyl)pyrrole (PP)

1-(*tert*-Butoxycarbonyl)-1*H*-pyrrole

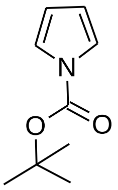

A solution of pyrrole (38.0 mL, 0.504 mol), di-*tert*-butylcarbonate (146.45 g, 0.671 mol), and DMAP (9.38 g, 0.077 mol) in a mixture of acetonitrile (375 mL) and triethylamine (200 mL) was stirred at r.t. for 21h. Water (125 mL) was added and the resulting mixture was left for 30 min with stirring. Then it was concentrated and the residue was partitioned between water and hexane. The water layer was washed with hexane and discarded. The combined organic layers were washed with brine, dried, and concentrated. The expected product was isolated by vacuum distillation (70-79 °C, 3.4-8.5 mbar) to give 1-(*tert*-Butoxycarbonyl)-1*H*-pyrrole (64.46 g, 0.386 mol, 77%) as a colourless oil.

1-(*tert*-Butoxycarbonyl)pyrrole-2-yl)boronic acid

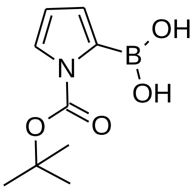

The reaction was carried out under anhydrous conditions. 2,2,6,6-Tetramethylpiperidine (50 mL, 0.296 mol) was dissolved in anhydrous THF (350 mL) under argon atmosphere. The reaction mixture was cooled to -78 °C and *n*-BuLi (2.5 M in hexanes, 120 mL, 1.298 mol) was added. The reaction mixture was then allowed to warm up to 0 °C, and then once again cooled to -78 °C. A solution of 1-(*tert*-Butoxycarbonyl)-1*H*-pyrrole (45.37 g, 0.271 mol) in anhydrous THF (40 mL) was added at such a rate, that the temperature does not exceed -60 °C. Then stirring at -78 °C was continued for 2h. $B(OMe)_3$ (50.0 mL, 0.448 mol) was added and the reaction mixture was allowed to warm up to room temperature. Then a mixture of

water (320 mL) and concentrated HCl (120 mL) was added. This mixture was extracted twice with ether. The organic layers were washed with brine, dried over anhydrous Na_2SO_4 and concentrated. The remaining solid was suspended in ether (200 mL) and warmed to reflux. The remaining solid was collected by filtration and dried on air to give 1-(*tert*-butoxycarbonyl)pyrrole-2-yl)boronic acid (35.71 g, 0.169 mol, 62%), as a yellowish solid.

1-(*tert*-Butoxycarbonyl)-2-(pyridin-2-yl)-1*H*-pyrrole

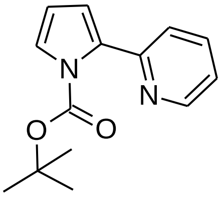

The mixture of THF (160 mL), water (160 mL), Na_2CO_3 (8.76 g), 1-(*tert*-butoxycarbonyl)pyrrole-2-yl)boronic acid (10.377 g; 0.0425 mol) and 2-bromopyridine (3.82 mL, 0.040 mol) was deoxygenated. Then $(Ph_3P)_4Pd$ (1.92 g, 0.0017 mol) was added under argon and the reaction mixture was stirred under reflux for 2h. Then it was cooled to room temperature and extracted twice with ether. The combined organic layers were dried and concentrated. The oily residue was chromatographed (hexanes/ethyl acetate 9/1 then 4/1) to collect the more polar of two products. 1-(*tert*-Butoxycarbonyl)-2-(pyridin-2-yl)-1*H*-pyrrole (6.238 g, 0.0255 mol, 64%) was isolated as a yellow, oily liquid.

2-(2’-pyridyl)pyrrole

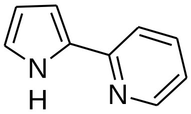

1-(*tert*-Butoxycarbonyl)-2-(pyridin-2-yl)-1*H*-pyrrole (6.238 g, 0.0255 mol) was dissolved in dichloromethane (15 mL) and trifluoroacetic acid (15 mL) was added. The reaction mixture was stirred at r.t. for 3h, poured into 5% water solution of Na_2CO_3 (400 mL), and extracted with ether. The organic layer was washed with water, dried over anhydrous Na_2SO_4, and concentrated. The remaining green solid was washed with *n*-pentane and the expected product was purified by sublimation. 2-(2’-pyridyl)pyrrole (1.422 g, 0.0099 mmol, 39%) was received as a white, crystalline solid.

molecules

References

1. Gorski, A.; Gawinkowski, S.; Luboradzki, R.; Tkacz, M.; Thummel, R.P.; Waluk, J. Polymorphism, Hydrogen Bond Properties, and Vibrational Structure of 1H-Pyrrolo[3,2-h]Quinoline Dimers. *J. At. Mol. Opt. Phys.* **2012**, *2012*, 1–11, doi:10.1155/2012/236793.
2. Krasnokutskii, S.N.; Kurbovskaya, L.N.; Shibanova, T.A.; Shabunova, V.P. Structure of 1H-pyrrolo[3,2-h]quinoline. *J. Struct. Chem.* **1991**, *32*, 106–110, doi:10.1007/BF00744952.
3. Noland, W.E.; Cole, K.P.; Britton, D. Five (1H-pyrrol-2-yl)pyridines. *Acta Crystallogr. Sect. C Cryst. Struct. Commun.* **2003**, *59*, o263–o267, doi:10.1107/S0108270103007042.

© 2021 by the authors. Submitted for possible open access publication under the terms and conditions of the Creative Commons Attribution (CC BY) license (http://creativecommons.org/licenses/by/4.0/).
